# *SLC2As* as diagnostic markers and therapeutic targets in LUAD patients through bioinformatic analysis

**DOI:** 10.3389/fphar.2022.1045179

**Published:** 2022-11-28

**Authors:** Yanli Zhang, Han Qin, Jing Bian, Zhanchuan Ma, Huanfa Yi

**Affiliations:** ^1^ Central Laboratory, The First Hospital of Jilin University, Changchun, China; ^2^ Key Laboratory of Organ Regeneration and Transplantation, Ministry of Education, Changchun, Jilin, China; ^3^ Echocardiography Department, The First Hospital of Jilin University, Changchun, China; ^4^ Department of Respiratory Medicine, The First Hospital of Jilin University, Changchun, China

**Keywords:** *SLC2As*, lung adenocarcinoma, diagnostic maker, therapeutic targets, glucose transporters

## Abstract

Facilitative glucose transporters (GLUTs), which are encoded by solute carrier 2A (*SLC2A*) genes, are responsible for mediating glucose absorption. In order to meet their higher energy demands, cancer cells are more likely than normal tissue cells to have elevated glucose transporters. Multiple pathogenic processes, such as cancer and immunological disorders, have been linked to GLUTs. Few studies, meanwhile, have been conducted on individuals with lung adenocarcinoma (LUAD) to evaluate all 14 *SLC2A* genes. We first identified increased protein levels of *SLC2A1*, *SLC2A5*, *SLC2A6*, and *SLC2A9 via* HPA database and downregulated mRNA levels of *SLC2A3*, *SLC2A6*, *SLC2A9*, and *SLC2A14* by ONCOMINE and UALCAN databases in patients with LUAD. Additionally, lower levels of *SLC2A3*, *SLC2A6*, *SLC2A9*, *SLC2A12*, and *SLC2A14* and higher levels of *SLC2A1*, *SLC2A5*, *SLC2A10*, and *SLC2A11* had an association with advanced tumor stage. *SLC2A1*, *SLC2A7*, and *SLC2A11* were identified as prognostic signatures for LUAD. Kaplan-Meier analysis, Univariate Cox regression, multivariate Cox regression and ROC analyses further revealed that these three genes signature was a novel and important prognostic factor. Mechanistically, the aberrant expression of these molecules was caused, in part, by the hypomethylation of *SLC2A3*, *SLC2A10*, and *SLC2A14* and by the hypermethylation of *SLC2A1*, *SLC2A2*, *SLC2A5*, *SLC2A6*, *SLC2A7*, and *SLC2A11*. Additionally, *SLC2A3*, *SLC2A5*, *SLC2A6*, *SLC2A9*, and *SLC2A14* contributed to LUAD by positively modulating M2 macrophage and T cell exhaustion. Finally, pathways involving *SLC2A1*/BUB1B/mitotic cell cycle, *SLC2A5*/CD86/negative regulation of immune system process, *SLC2A6*/PLEK/lymphocyte activation, *SLC2A9*/CD4/regulation of cytokine production might participate in the pathogenesis of LUAD. In summary, our results will provide the theoretical basis on *SLC2As* as diagnostic markers and therapeutic targets in LUAD.

## Introduction

Lung adenocarcinoma (LUAD), the most prevalent NSCLC subtype, has a high morbidity and mortality rate that makes it a global public health concern (Liu et al., 2015). Even though traditional treatment approaches involving curative resection, targeted therapy, or immunotherapy have seen enormous advancements in recent years, it is crucial to comprehend the molecular pathogenesis and etiology of LUAD to develop new prognostic and therapeutic targets for LUAD (Tong et al., 2018).

Tumor cells exhibit a distinct metabolism from normal cells, converting to higher glycolysis demands (the Warburg effect) and glucose intake for ATP synthesis to meet their energy needs. Facilitative glucose transporters (GLUTs), which are encoded by 14 *SLC2A* genes, are responsible for mediating this process. Based on function and sequence similarity, these transporters can be divided into three groups. Additionally, they differ in terms of tissue location and their affinity for the substrate (glucose and other hexoses like fructose). GLUT-1-4 and GLUT-14 are classified as Class 1 GLUTs, whereas GLUT-5, GLUT-7, GLUT-9, and GLUT-11 are Class 2 GLUTs. GLUT-6, GLUT-8, GLUT-10, GLUT-12, and GLUT-13 (H(+)-myo-inositol transporter HMIT) are classified as Class 3 GLUTs (Cura and Carruthers, 2012). Several clinical diseases, including cancer and autoimmune illness, are associated with *SLC2As* (Hu et al., 1999; Macintyre et al., 2014; Yin et al., 2015; Jodeleit et al., 2018; Tilekar et al., 2020a). Previous research has determined the upregulation of *SLC2As* and their roles in certain malignancies. Additionally, numerous types of researches have focused on GLUT-inhibitors to slow the growth of tumors (including breast cancer, osteosarcoma, and NSCLC) and other proliferative illnesses (Sanli et al., 2010; Storozhuk et al., 2012; Storozhuk et al., 2013; Hardie and Ashford, 2014). GLUT inhibitors have shown encouraging results in the treatment of tumors, particularly when combined with other chemotherapy, radiation, immunotherapy, pathway-specific oncogenic-targeted medicines, other anti-metabolic, or epigenetic medications (Granchi et al., 2016). However, off-targets and low potency, which is a result of cancer cell potency to adopt alternative strategies for glucose supply, still exist (Tilekar et al., 2020b). Additionally, in specific tumor forms, glucose uptake does not coincide with GLUT-1 expression, suggesting the participation of other transporters. To put it another way, GLUT-1 may have an impact *via* working with other GLUTs. All these reports serve as a reminder that to evaluate the transport members’ roles in a particular cancer type, they must be considered as a whole. The same is true for LUAD; we could only reliably anticipate outcomes and identify patients who would respond better to particular GLUT inhibitors if we thoroughly evaluated the metabolic profiles in LUAD.

To explore the molecular landscape of LUAD patients, we first evaluated the DNA methylation, mRNA, and protein levels of 14 *SLC2As* in this study. Then, to better grasp the potential of *SLC2As* as diagnostic biomarkers and prognosis indicators of LUAD, we further investigated the relationship between the expressions of 14 *SLC2As* and clinical data. A theoretical foundation for *SLC2As* as therapeutic targets in LUAD is provided by our exploration of the molecular mechanisms that *SLC2As* contribute to the pathophysiology of LUAD.

## Materials and methods

### Ethic statement

Samples of tumor tissues and adjacent tissues were taken from 6 LUAD patients from The First Hospital of Jilin University. Each participant gave their written informed consent in accordance with the principles of the Declaration of Helsinki. Additionally, the hospital’s institutional ethics committee gave its approval for this investigation.

### Transcriptional level of *SLC2As* analysis *via* ONCOMINE and UALCAN database

ONCOMINE (Rhodes et al., 2004) is the largest integrated database mining platform and cancer gene chip database. The difference in *SLC2A* mRNA expression between LUAD patients and healthy lung tissues was examined. The *p* threshold was set at 1E-4 and the fold change threshold was set at 2. UALCAN (Chandrashekar et al., 2017) is a comprehensive online analytic tool that uses RNA-seq and clinical data from 31 different cancer types from the TCGA database. The *SLC2As* mRNA expression profiles and DNA methylation levels in individuals with LUAD were compared to those in healthy tissues using the UALCAN database. Additionally, the UALCAN database was used to evaluate the relationship between the expression of *SLC2As* and clinicopathologic characteristics. The *p*-value was calculated using Student’s *t*-test, and the threshold was set at 0.05.

### Analysis of *SLC2As* protein levels using the human protein altas database

The Human Protein Altas (HPA) (Uhlén et al., 2015) is a website that allows users to investigate each protein’s expression level and location in 20 cancer types that are extremely prevalent. We employed HPA to examine how the expression of GLUTs varied between LUAD patients and healthy lung tissue.

### Construction and evaluation of the prognostic nomogram

RNA-sequencing expression (level 3) profiles and corresponding clinical information for LUAD were downloaded from the TCGA dataset (https://portal.gdc.com). To choose the appropriate terms for the nomogram, univariate and multivariate cox regression analysis were used. With the use of the R program “forestplot,” the forest was utilized to display the *p*-value, HR, and 95% confidence interval for each variable (Xiong et al., 2020). Based on the outcomes of the multivariate cox proportional hazards analyses a nomogram was created to forecast the overall recurrence over the next 1, 2, 3, 5 year. The nomogram offered a graphical depiction of the elements that may be used to determine a patient’s unique risk of recurrence based on the points assigned to each risk factor using the “rms” R program (Jeong et al., 2020; Xiong et al., 2020).

### Construction and evaluation of the prognostic risk model of *SLC2As*

Kaplan-Meier plotter (Győrffy et al., 2013) was employed to examine the relationship between the transcriptional levels of *SLC2As* and the survival of LUAD patients. *p* ≤ 0.05 was used to define the statistical significance level.

From the TCGA dataset, RNA-sequencing expression (level 3) profiles and associated clinical data for LUAD were obtained (https://portal.gdc.com). Univariate Cox regression was used to assess the 14 genes in question, and candidate genes were chosen if they satisfied the screening requirement of *p* < 0.05. Following that, we used the R software’s “glment” package to perform LASSO regression on high-dimensional data to identify the most effective prognostic factors (Xu et al., 2021). Three genes were chosen, and a risk score was also computed for each of them. Based on the median expression of *SLC2A* genes, patients were split into high-risk and low-risk groups. Using the KM survival approach, the association between *SLC2A* genes and survival rates was examined. The *p*-value of KM survival curves was determined using log-rank testing. The receiver operating characteristic (ROC) curve was created to evaluate the model’s accuracy. To find LUAD prognostic variables, univariate and multivariate Cox regression analyses were performed. R (foundation for statistical computing 2020) version 4.0.3 was used to implement all the analysis techniques and R packages (Supplementary Material S1). A *p*-value of 0.05 was regarded as statistically significant.

### Co-expression genes of *SLC2As* mRNA in lung adenocarcinoma

We wanted to further explore the underlying mechanism *SLC2As* regulating LUAD. Thus, we analyzed the co-expression profiles *in* LUAD using the cBioPortal database (Cerami et al., 2012). The top 50 positively co-expressed genes and 50 negatively co-expressed genes were selected based on Spearman’s correlation.

### Gene ontology (GO) and kyoto encyclopedia of genes and genomes (KEGG) enrichment analysis

Metascape (Zhou et al., 2019) is a web-based database that incorporates over 40 different gene functions. The co-expressed genes were enriched using GO and KEGG analyses. We defined minimum overlap as 3, minimum enrichment as 1.5, and *p* ≤ 0.05 as significant.

### Protein-protein interaction (PPI) networks of the member of *SLC2As via* Cytoscape

Cytoscape (Smoot et al., 2011), a software focusing on open source network visualization and analysis, provides the basic function layout and query network. The Search Tool for the Retrieval of Interacting Genes/Proteins (STRING) database was used to build a PPI network to evaluate the potential PPI of co-expressed genes (Szklarczyk et al., 2021). PPI pairs with a minimum interaction value of 0.4 were chosen, and Cytoscape 3.8.2 was used to display the network. According to the degree score of each gene node, the CytoHubba plugin for Cytoscape approved the top 10 core genes.

### The association between *SLC2As* and immune cells infiltration in patients with LUAD

TIMER (Li et al., 2017) investigates in detail the immune infiltration status of different cancer types. In our study, the correlation between the immune cell marker genes and *SLC2As* in the correlation module was used to evaluate the relationship between *SLC2As* expressions and the infiltration of immune subtypes in LUAD patients. All the markers’ genes with coefficients greater than 0.35 were chosen.

### Quantitative polymerase chain reaction

We reanalyzed our prior projected *SLC2A3*, *SLC2A6*, *SLC2A9*, and *SLC2A14* expression level results, including paired tumor and surrounding tissues from 6 primary LUAD patients diagnosed in The First Hospital of Jilin University from June 2021 to June 2022, to see if the mRNA expressions of *SLA2As* are parallel to those in clinical samples. A TRIzol-based (Invitrogen) technique was used to extract the RNA, and RT EasyTM (with gDNase) was used to create complementary deoxyribonucleic acid (FORE GENE). On an ABI StepOnePlus machine from Applied Biosystems, using the Power q-PCR SYBR Green Mix, all PCRs were run in triplicate (Yeasen). Using the Cttechnique, the relative mRNA expression of several genes was measured. One of the housekeeping genes was GAPDH. The additional information included a list of the primer sequences (Comate Bioscience) (Table 1). We calculated relative gene expression levels with 2^−ΔΔCT^, visualizing data with Graphpad 9.0.

**TABLE 1:** The primer sequence of *SLC2A3*, *SLC2A6*, *SLC2A9*, *SLC2A14*.

	Forward primer (5′-3′)	Reverse primer (5′-3′)
Human-*SLC2A3*	GGTCGCTTGGTTATTGGC	ACCGCTGGAGGATCTGCT
Human-*SLC2A6*	GGT​GTA​CGT​GTC​TGA​GAT​TGC	CCT​GGA​TCT​GCT​CGA​ACT​CC
Human-*SLC2A9*	CAA​TAG​ACC​CAG​ACA​CTC​TGA​CT	TCT​TCA​CAA​TTA​ACG​TCC​CCA​C
Human-*SLC2A14*	GAG​ATG​GAC​AAC​AGA​CAG​AAT​GT	AAA​CAG​GCC​ACA​AAC​GAC​C

### Stastical analysis

Univariate survival analysis with Kaplan-Meier and log-rank tests. Multifactor survival analysis was conducted using COX regression models. By calculating the area under the curve using the pROC software package, we were able to assess the specificity and sensitivity of *SLC2As* in prognosis of LUAD. The continuous variables were compared using independent *t*-tests. Categorical data were tested with chi-square tests. Statistical significance was defined as a two-sided *p*-value < 0.05. All data processing was done in R4.0.3 software.

## Results

### Functional annotation of *SLC2As*

**FIGURE 1:**
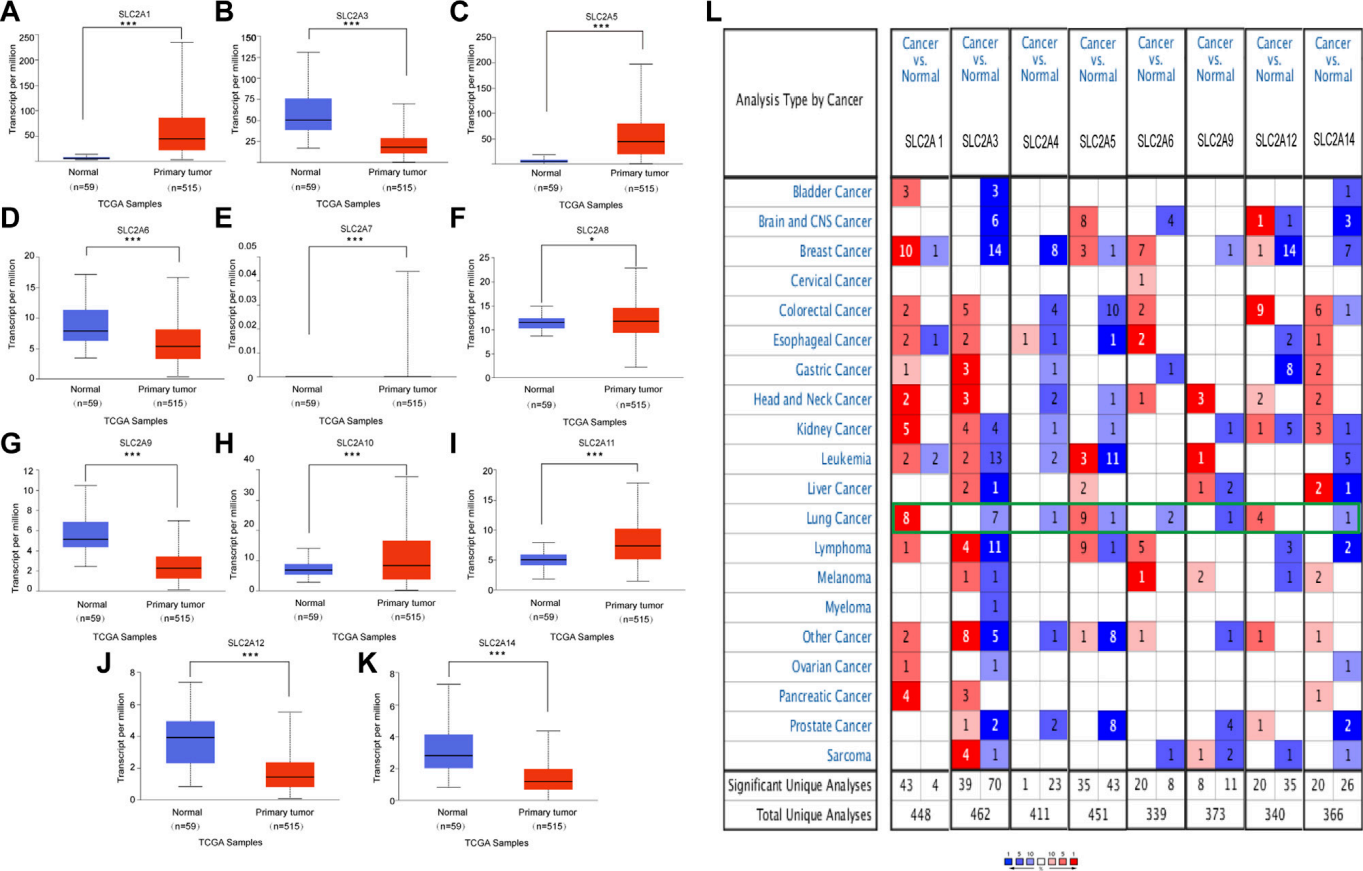
mRNA expression of distinct *SLC2As* in LUAD tissues and adjacent normal lung tissues from UALCAN and ONCOMINE database. **(A–K)** (UALCAN) mRNA expressions of *SLC2A1*, *SLC2A5*, *SLC2A7*, *SLC2A8*, *SLC2A10*, *SLC2A11* were over-expressed while *SLC2A3*, *SLC2A6*, *SLC2A9*, *SLC2A12*, *SLC2A14* were downregulated in primary LUAD tissues compared to normal lung tissues. ***p < 0.001, **<0.01, *<0.05. **(L)** Transcriptional expression of *SLC2As* in 20 different types of cancer diseases (ONCOMINE database). Difference of transcriptional expression was compared by Students’ *t*-test. Cut-off of *p* value and fold change were as following: *p* value: 0.05, fold change: 2.0, gene rank: 10%, data type: mRNA (ONCOMINE).

**FIGURE 2:**
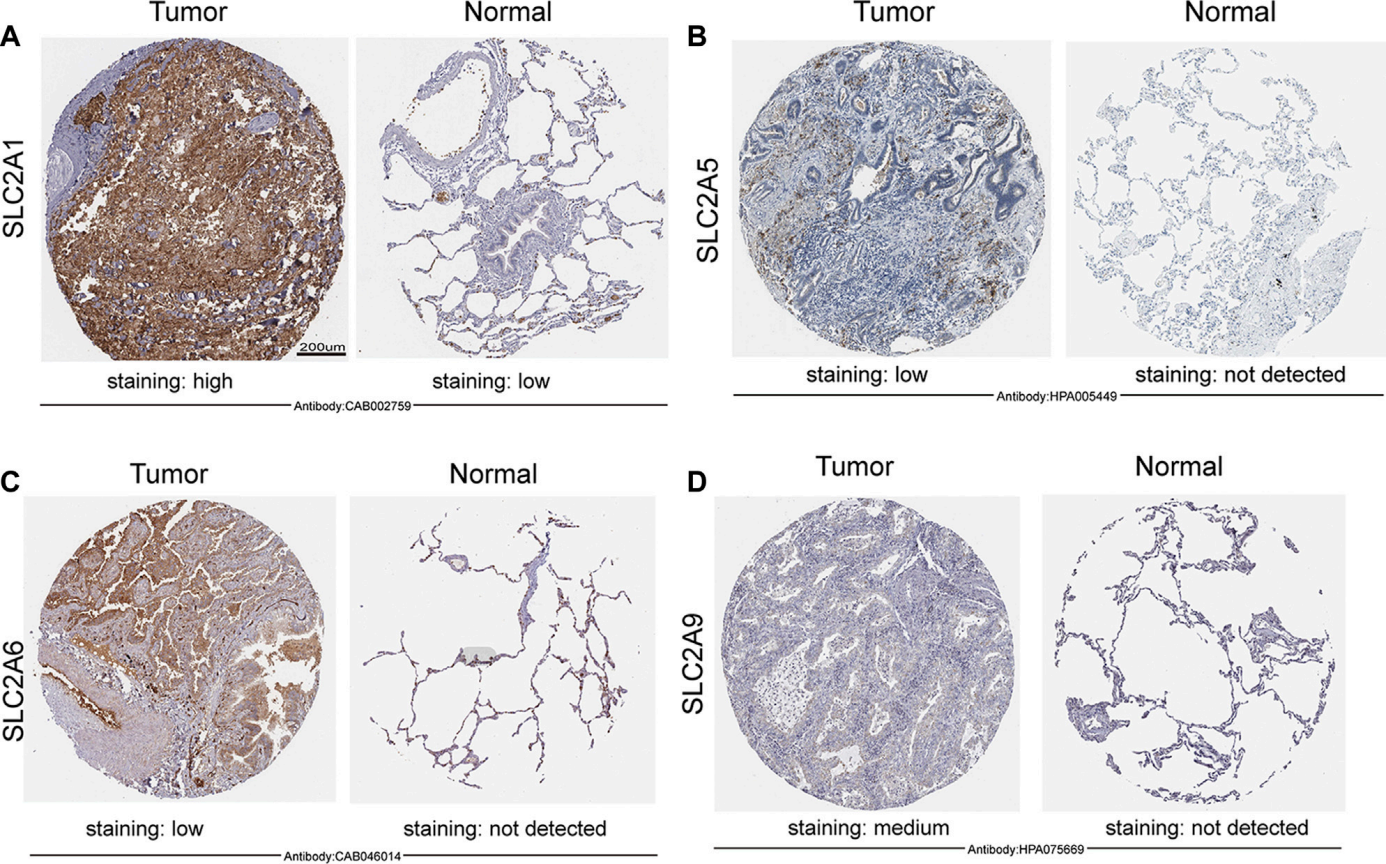
Expression of *SLC2As* protein in LUAD tissues and adjacent lung tissues (HPA). Scale bar, 200 µm. **(A)** Lung tissues displayed low GLUT 1 expression while LUAD tumors showed high protein expressions of it. **(B–D)** GLUT 5, GLUT 6, GLUT 9 were not expressed in normal lung tissues, while low or medium protein levels were expressed in LUAD tissues.

The overview of the research design is present in Supplementary Material S2; Figure 1. First and foremost, we are interested in the functional annotation of *SLC2As*. As shown in Supplementary Material S2; Figure 2, Gene ontology (GO) and Kyoto Encyclopedia of Genes and Genomes (KEGG) analysis in Metascape database were employed to analyze the function and pathways *SLC2A* family members participated in. Biological processes (BP) such as GO: 1904659 (glucose transmembrane transport), GO:0070837 (dehydroascorbic acid transport), GO: 0098704: (carbohydrate import across plasma membrane) and GO: 0032868 (response to insulin) were closely correlated with *SLC2As* (Supplementary Material S2; Figure 2A). Cellular components (CC), involving GO: 0016324 (apical plasma membrane), GO: 0098793 (presynapse), GO: 0048471 (perinuclear region of cytoplasm) (Supplementary Material S2; Figure 2B) and molecular functions (MF) such as GO: 0005355 (glucose transmembrane transporter activity), GO: 0033300 (dehydroascorbic acid transmembrane transporter activity), GO: 0005353 (fructose transmembrane transporter activity), GO: 0015295 (solute: proton symporter activity) were enriched for *SLC2As* (Supplementary Material S2; Figure 2C). KEGG pathway analysis indicates pathway 04931: *SLC2As* and insulin resistance were linked (Supplementary Material S2; Figure 2D). Additionally, *SLC2As* were linked to many diseases, such as intestinal atresia, pregnancy-related diabetes, hypouricemia, Lesch-Nyhan syndrome, gestational diabetes, arterial tortuosity syndrome, meningomyelocele, and addictive behavior (Supplementary Material S2; Figure 2E).

### Abnormal expressions of *SLC2As* in patients with LUAD

We initially determined the mRNA and protein expression of *SLC2As* using the ONCOMINE, UALCAN, and HPA databases to investigate the diagnostic and therapeutic relevance of *SLC2As* in LUAD patients. In the UALCAN database, we first verified the mRNA expression of SLC2A family members, The findings in Figures 1A–K demonstrated that LUAD tissues had lower levels of *SLC2A3*, *SLC2A6*, *SLC2A9*, *SLC2A12*, and *SLC2A14* than normal lung tissues and higher levels of *SLC2A1*, *SLC2A5*, *SLC2A7*, *SLC2A8*, *SLC2A10*, and *SLC2A11*. The ONCOMINE database, which was different from UALCAN database, was used to quantify the mRNA expressions of 14 *SLC2A* genes in 20 different types of malignancies and compare them to normal tissues. LUAD tumor tissues showed higher mRNA expressions of *SLC2A1*, *SLC2A5*, and *SLC2A12* and lower expressions of *SLC2A3*, *SLC2A4*, *SLC2A6*, *SLC2A9*, and *SLC2A14* (Figure 1L). Together, these findings demonstrated that individuals with LUAD had transcriptionally upregulated levels of *SLC2A1*, *SLC2A5*, and downregulated levels of *SLC2A3*, *SLC2A6*, *SLC2A9*, and *SLC2A14*. Using the HPA database, we attempted to identify the protein levels of *SLC2As* in LUAD. According to the findings in Figure 2, GLUT-1 protein expression was shown to be low in lung tissues and high in LUAD tissues (Figure 2A). Additionally, while low or medium protein levels were expressed in LUAD tissues, GLUT-5, GLUT-6, and GLUT-9 were not detected in normal lung tissues (Figures 2B–D). In summary, GLUT-1, GLUT-5 GLUT-6, and GLUT-9 protein levels were overexpressed in patients with LUAD.

### Correlation between *SLC2As* family mRNA expressions and clinical tumor stage

**FIGURE 3:**
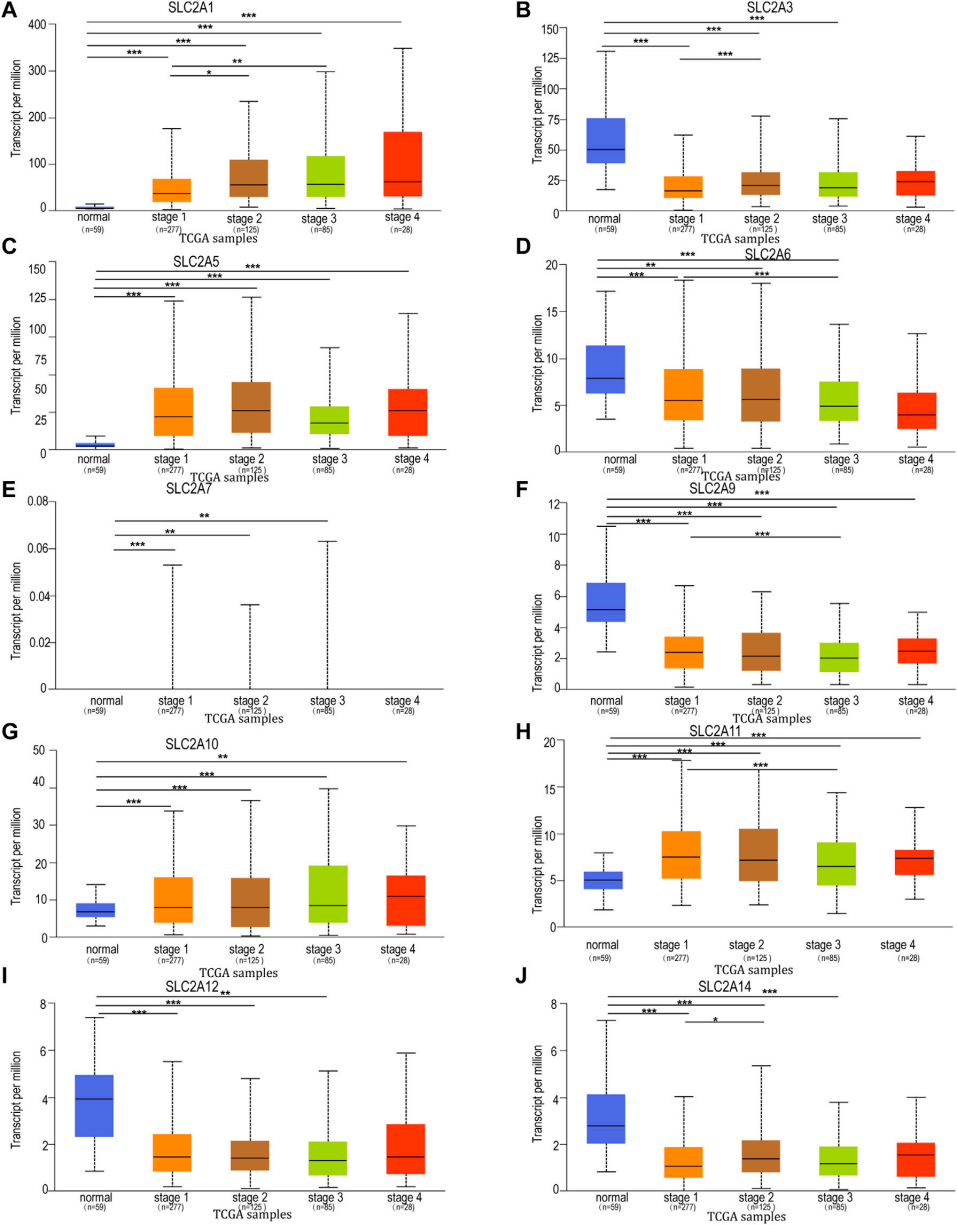
Relationship between mRNA expression of distinct *SLC2As* and individual cancer progression of LUAD patients. mRNA expressions of *SLC2As* were remarkably correlated with patients’ individual cancer stages. **(A,C,G,H)** Patients who were in more advanced stages tended to express higher mRNA expression of *SLC2A1*, *SLC2A5*, *SLC2A10*, *SLC2A11*. The highest mRNA expressions of, *SLC2A5*, *SLC2A10*, *SLC2A11* were found in stage 4. **(B,D,F,I,J)** Patients who were in more advanced stages tended to express lower mRNA expression of *SLC2A3*, *SLC2A6*, *SLC2A9*, *SLC2A12* and *SLC2A14*. **(A–I)** In addition, *SLC2A1*, *SLC2A3*, *SLC2A5*, *SLC2A6*, *SLC2A7*, *SLC2A9*, *SLC2A10*, *SLC2A11*, *SLC2A12*, *SLC2A14* expressions were different in stage 1 from that in normal group. *p < 0.05, **p < 0.01, ***p < 0.001.

To further explore the relation of *SLC2As* with clinical characters, the association between *SLC2As* expressions and patients’ cancer stages in LUAD patients by UALCAN database was then investigated. As shown in Figure 3, *SLC2A1*, *SLC2A5*, *SLC2A10*, and *SLC2A11* levels tended to be higher in patients with advanced tumor stages, which shows that these molecules may be involved in the development of malignancies in LUAD patients. Furthermore, the highest expression of *SLC2A1*, *SLC2A5*, *SLC2A10*, and *SLC2A11* was observed in stage 4, whereas *SLC2A5* and *SLC2A11* expression in stage 3 was lower than in stage 2. This suggests that the difference in sample sizes between stage 2 (*n* = 125) and stage 3 (*n* = 85) may be a reasonable explanation for the contradictory results. Furthermore, *SLC2A3*, *SLC2A6*, *SLC2A9*, *SLC2A12*, and *SLC2A14* levels were lower in patients with advanced tumor stages, suggesting that these genes might act as a barrier to the progression of LUAD. Interestingly, the expression levels of *SLC2A1*, *SLC2A5*, *SLC2A10*, *SLC2A11*, *SLC2A3*, *SLC2A6*, *SLC2A9*, *SLC2A12*, and *SLC2A14* differed in stage I LUAD tissues compared to normal tissues, suggesting that these molecules could act as non-invasive biomarkers for LUAD early detection. All of these findings open the door for these *SLC2A* genes to be used as possible biomarkers for the early identification and precise stratification of LUAD patients.

### Construction and evaluation of *SLC2As*-related prognostic model

**FIGURE 4:**
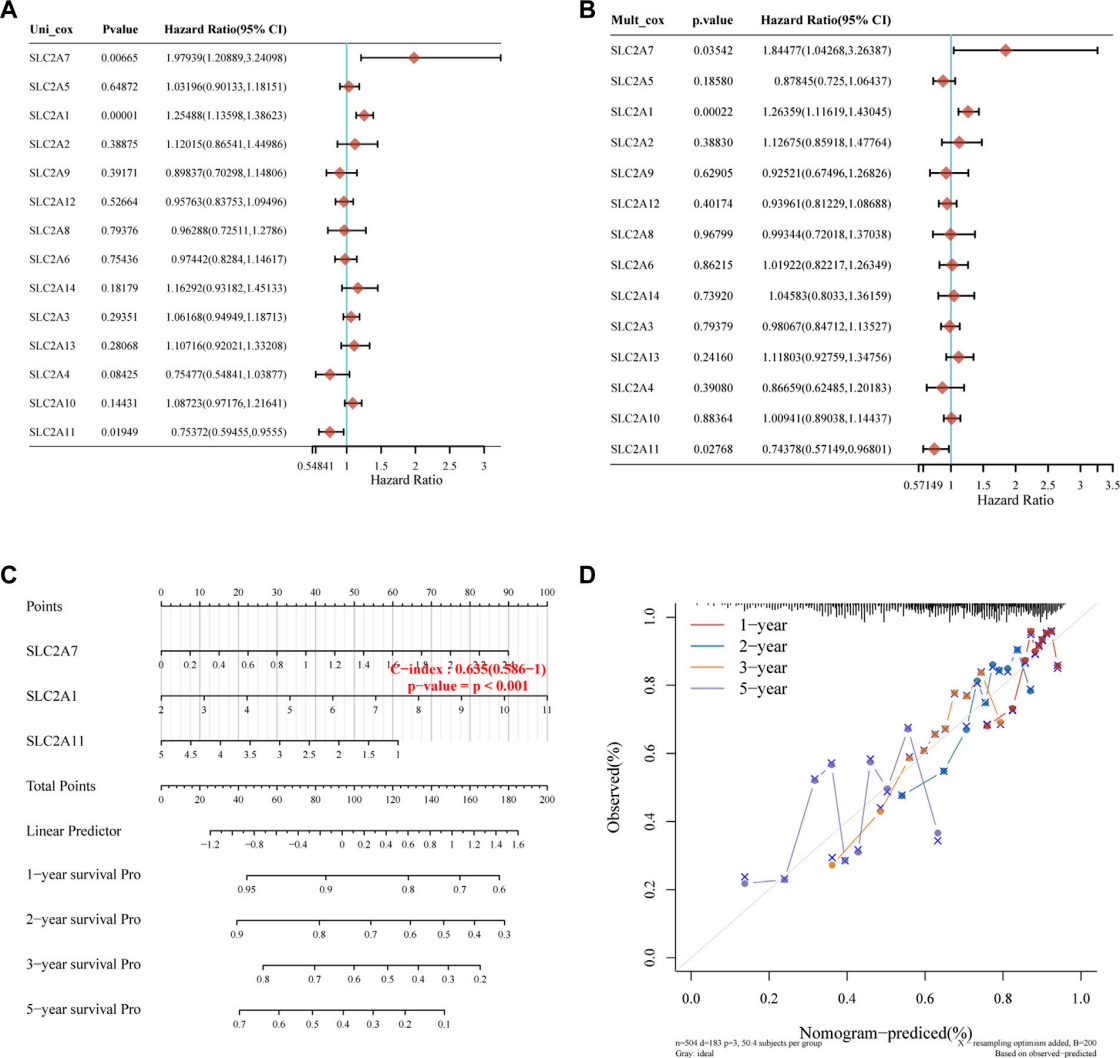
Construction of the nomogram model. **(A,B)** Forest plots for hazard ratios of survival-associated SLC2As in LUAD through univariate and multivariate Cox regression tests. *SLC2A1*, *SLC2A7*, and *SLC2A11* were independent predictors of prognosis according to univariate and multivariate Cox regression analysis of the *p* value, risk coefficient (HR), and confidence interval of *SLC2As*. **(C)** Nomogram can forecast the overall survival of LUAD patients at 1, 2, 3, and 5 years. **(D)** Calibration curve for the overall survival nomogram model in the discovery group. The blue line, red line, and orange line indicate the 1-year, 2-year, 3-year, and 5-year of the observed nomogram, respectively. This suggests that the prognostic model could successfully predict the prognosis of LUAD. The dashed diagonal line represents the ideal nomogram.

**FIGURE 5:**
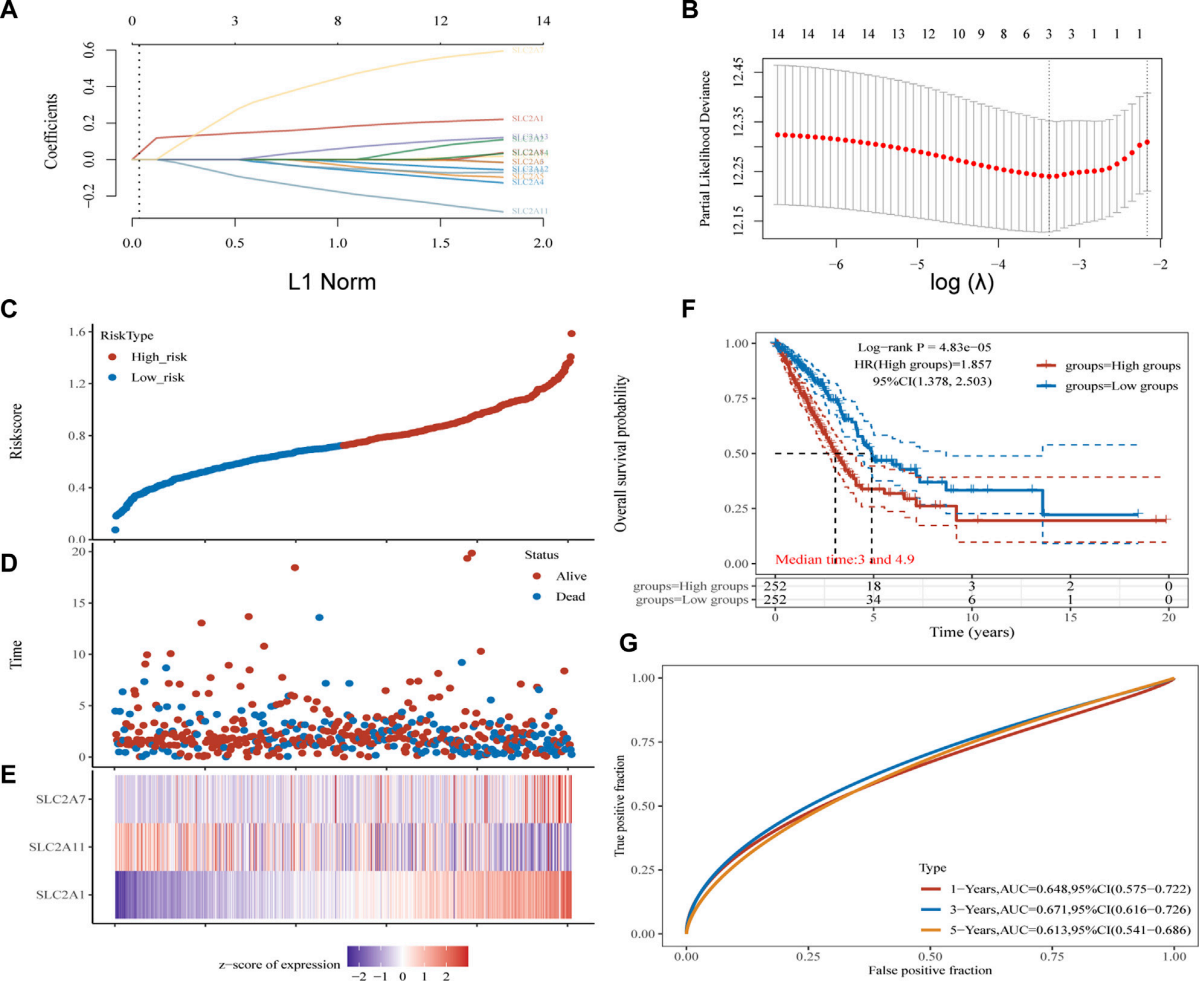
Construction and validation of *SLC2As*-related signature. **(A)** The coefficients of *SLC2As* are shown by lambda parameter. **(B)** Partial likehood versus log(λ) were manifested using LASSO model. **(C–E)** The Riskscore, survival time and survival status of LUAD. **(C)** The top scatterplot represents the Riskscore from low to high. Blue represents low risk group and red represents high risk group. **(D)** The middle scatter plot distribution represents the Riskscore of different samples correspond to the survival time and survival status. **(E)** The bottom heatmap is the gene *SLC2A1*, *SLC2A7*, *SLC2A11* expression from the signature. **(F)** Kaplan-Meier survival analysis of the risk model from dataset, comparison among high and low group was made by log-rank test. HR = 1.857 indicates the gene set is a risk factor. The median survival time (LT50) for the high group and low group is 3 and 4.9 years, respectively, and the HR (95% CI) ranges from 1.378 to 2.503.**(G)** The ROC curve and AUC of the gene set for 1,3, and 5 year (64.8%, 67.1%, 61.3%).

Kaplan-Meier plotter online tool was used to predict the value of SLC2As in gauging the prognosis of LUAD. Lower mRNA expressions of *SLC2A1*, *SLC2A2*, *SLC2A3*, *SLC2A4*, *SLC2A5*, *SLC2A9*, *SLC2A14* and higher mRNA level of *SLC2A10*, *SLC2A12*, *SLC2A13* were significantly associated with favorable Overall Survial (OS) of LUAD (Supplementary Material S2; Figure 3). Furthermore, we ran univariate and multivariate Cox regression tests to see if the risk model of 14 SLC2As had distinct prognostic features for LUAD. *SLC2A1*, *SLC2A7*, and *SLC2A11* were chosen as independent predictors for survival in LUAD patients in univariate Cox regression analysis, with *SLC2A1*, *SLC2A7* serving as risk factors and *SLC2A11* as a favorable factor in LUAD (Figure 4A). *SLC2A1*, *SLC2A7*, and *SLC2A11* were also statistically significant in multivariate Cox regression analysis (Figure 4B), suggesting that these three genes may be the reliable predictors in LUAD. A nomogram based on *SLC2A1*, *SLC2A7*, and *SLC2A11* to forecast the 1, 2, 3, and 5 year overall recurrence was created (Figure 4C). The observed vs. predicted rates of the 1-, 2-, and 3-year OS showed excellent consistency in the correlation plots (Figure 4D). While attempting to uncover predictive gene sets for LUAD using the LASSO approach (Zhang et al., 2020), *λ* was chosen when the minimum sum of squared residuals was found. We ultimately discovered three genes, including *SLC2A1*, *SLC2A7*, and *SLC2A11* for subsequent multivariate analysis (Figures 5A,B). The risk score of 3 genes was also calculated for further univariate and multivariate Cox regression analyses. The hazard ratio between the low-expression sample and the high-expression sample is represented as HR (High risk). Based on the integrated model with cutoff values at the median expression of the three candidate genes, patients were categorized into high-risk and low-risk groups. The selected dataset’s risk score, survival period, and survival status. Risk score is shown in the top scatterplot, ranging from low to high (Figure 5C). The risk score of various samples, which corresponds to survival time and survival status, is represented by the scatter plot distribution (Figure 5D). The gene expression from the signature is displayed in the bottom heatmap. *SLC2A1* and *SLC2A7* levels that were higher and *SLC2A11* levels that were lower indicated significant risk in LUAD patients (Figure 5E). The risk model from the dataset underwent a Kaplan-Meier survival analysis, and a log-rank test was used to compare the outcomes of the various groups. Compared to the low-risk group, the high-risk group consistently had a poor prognosis (Figure 5F). By using ROC curves, we also compared the prognostic effect of risk factors. Areas under the curve (AUC) for 1-year OS, 3-year OS and 5-year OS were 64.8%, 67.1%, and 61.3%, respectively (Figure 5G), demonstrating that the combination of *SLC2A1*, *SLC2A7*, and *SLC2A11* might serve as a marker of prognosis of LUAD.

### Methylation levels of *SLC2As* in LUAD patients

**FIGURE 6:**
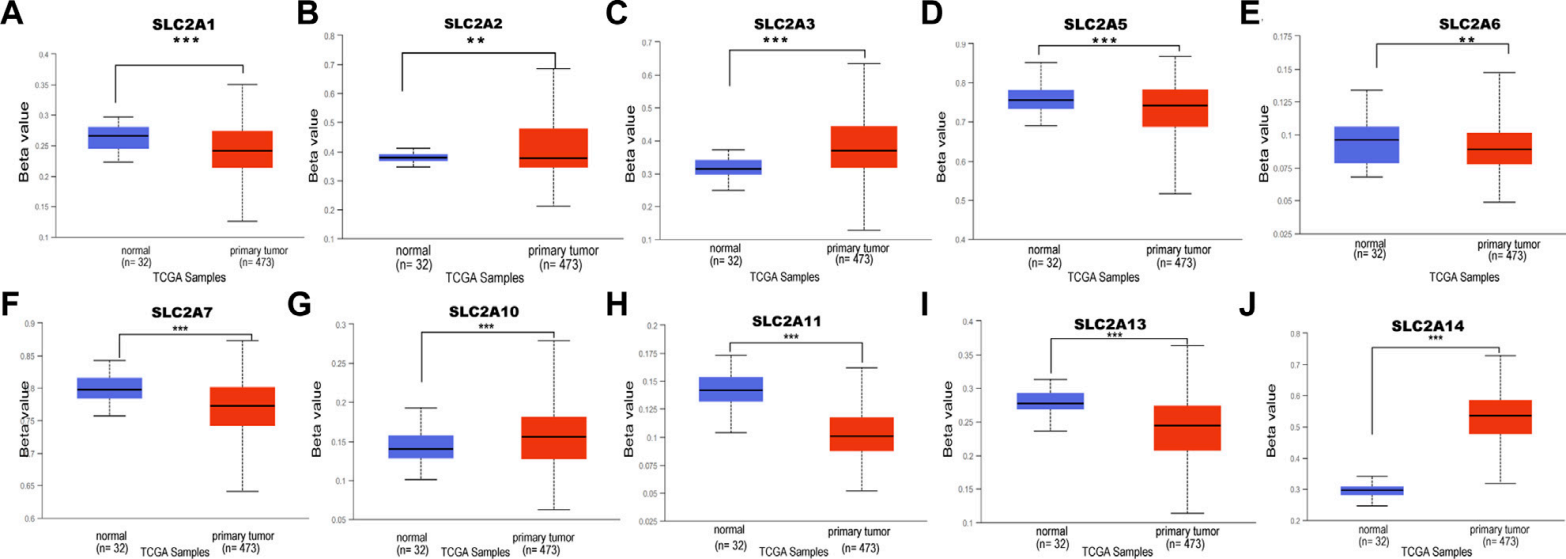
DNA methylation level of distinct SLC2As in LUAD tissues and adjacent normal lung tissues (UALCAN). Hypomethylation of *SLC2A1*, *SLC2A2*, *SLC2A5*, *SLC2A9*, *SLC2A11* **(A,B,D–F,H,I)** and hypermethylation of *SLC2A3*, *SLC2A10*, *SLC2A14* **(C,G,J)** were found in primary LUAD tissues compared with those in normal lung tissues. ***p < 0.001, **<0.01, *<0.05.

The upstream factor that dysregulates the expression of SLC2A was then identified. DNA methylation, one type of epigenetic change, involves adding a methyl group to the cytosine’s C5 position to create 5-methylcytosine. This occurs primarily at CG and CH (CH = CA, CT, and CC) locations (Wang et al., 2021). It has been suggested that DNA methylation has a role in a variety of biological processes, particularly the genesis of cancer. Next, we attempted to investigate how DNA methylation by *SLC2As* affects LUAD. According to data from the UALCAN database, LUAD patients’ tumor tissues had hypomethylation of *SLC2A1*, *SLC2A2*, *SLC2A5*, *SLC2A6*, *SLC2A7*, *SLC2A11* and hypermethylation of *SLC2A3*, *SLC2A10*, and *SLC2A14* compared to normal tissues (Figure 6).

### Construction of a network of *SLC2As* and co-expressed genes and identification of potential “hub” genes

**FIGURE 7:**
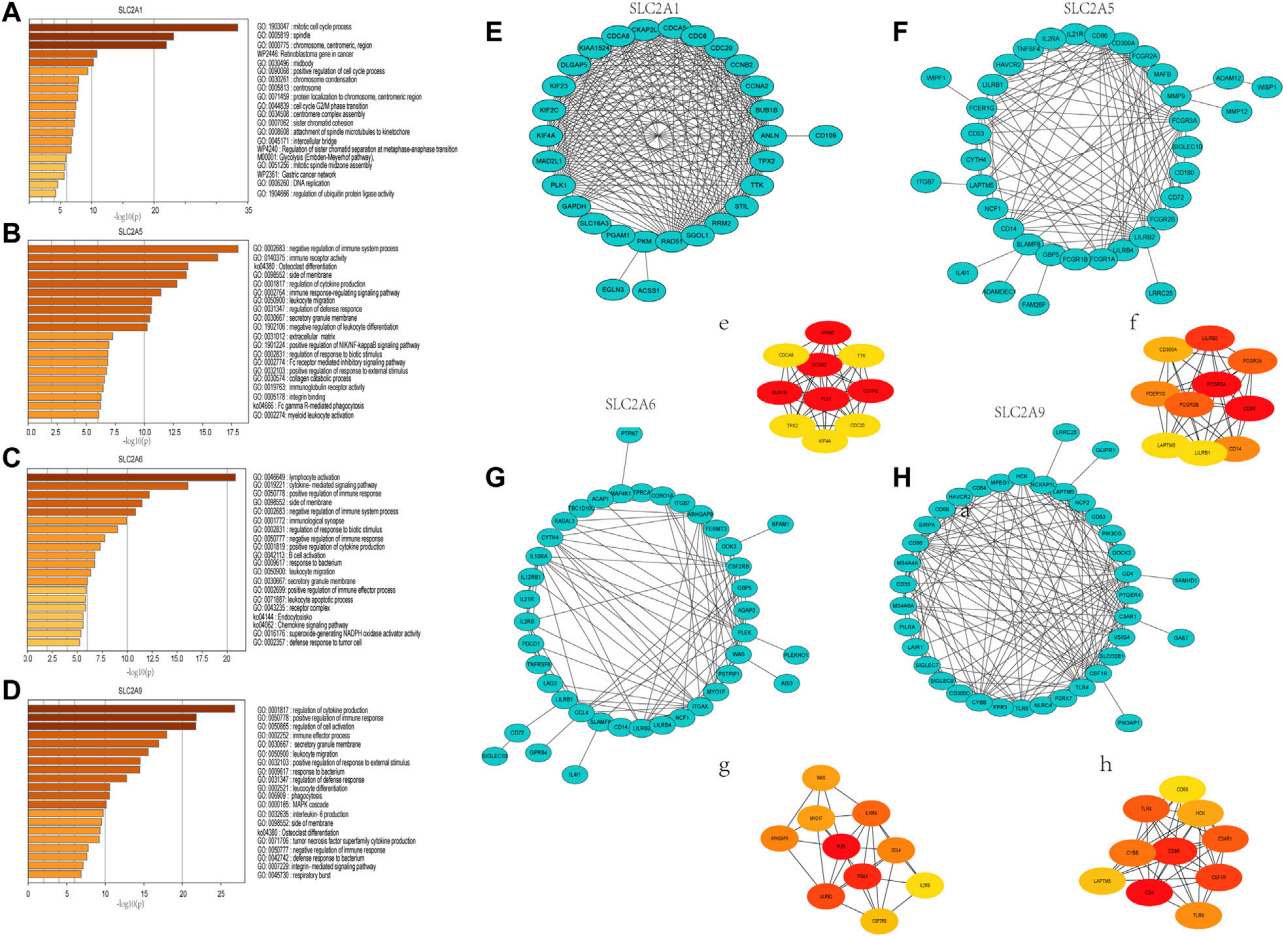
Gene Ontology (GO) annotations and Kyoto Encyclopedia of Genes and Genomes (KEGG) pathway analyses of genes that ranked top 100 co-expressed genes with *SLC2A1*, *SLC2A5*, *SLC2A6*, *SLC2A9*. **(A)** *SLC2A1*. **(B)** *SLC2A5*. **(C)** *SLC2A6*. **(D)** *SLC2A9*. Construction of an interaction network with *SLC2As* top 100 correlated genes and identification of potential hub genes. **(E–H)** PPI network of *SLC2A1*, *SLC2A5*, *SLC2A6*, and *SLC2A9* co-expressed genes. (e–h) The top 10 hub genes identified in the PPI network.

The functions and pathways in which *SLC2As* are involved in LUAD patients will next be further clarified. We performed GO and KEGG enrichment analyses to investigate the biological functions of the co-expression profiles of *SLC2A1*, *SLC2A5*, *SLC2A6*, and *SLC2A9* in LUAD patients (whose protein expression level was different in LUAD from that in normal tissues). The Metascape database was enriched for the top 100 genes (Supplementary Material S3) substantially linked with *SLC2A1*, *SLC2A5*, *SLC2A6*, and *SLC2A9* (according to *p* value) (Figures 7A–D). The genes that co-express *SLC2A1* were primarily enriched in the centromeric region of the chromosome and were grouped during the mitotic cell cycle process. *SLC2A5*-coexpressed genes were particularly rich in those that negatively regulated immune system function, immunological receptor activation, and osteoclast differentiation. Lymphocyte activation, cytokine-mediated signaling pathways, and the positive modulation of immune response were among the gene functions enriched in *SLC2A6* co-expression. The regulation of cytokine production, the positive regulation of immune response, and the regulation of cell activation were all enriched in the genes co-expressed with *SLC2A9*. According to the degree score of each gene node, the CytoHubba plugin for Cytoscape selected the top 10 core genes (Figures 7E–H, e–h). The core gene BUB1B (e), which was engaged in the mitotic cell cycle process and might be the most significant gene among those connected with *SLC2A1*, had the highest degree score. CD86 (f), a gene involved in the negative regulation of immune system activity, was the most significant gene for *SLC2A5*. The gene PLEK (g), which was involved in lymphocyte activation, was the most significant gene for *SLC2A6*. The CD4 (h) gene, which controlled cytokine production, was the most significant gene for *SLC2A9*. These results suggest that *SLC2As* are implicated in LUAD through orchestrating immune cells.

### *SLC2As* expressions are correlated with infiltration of immune cells

**FIGURE 8:**
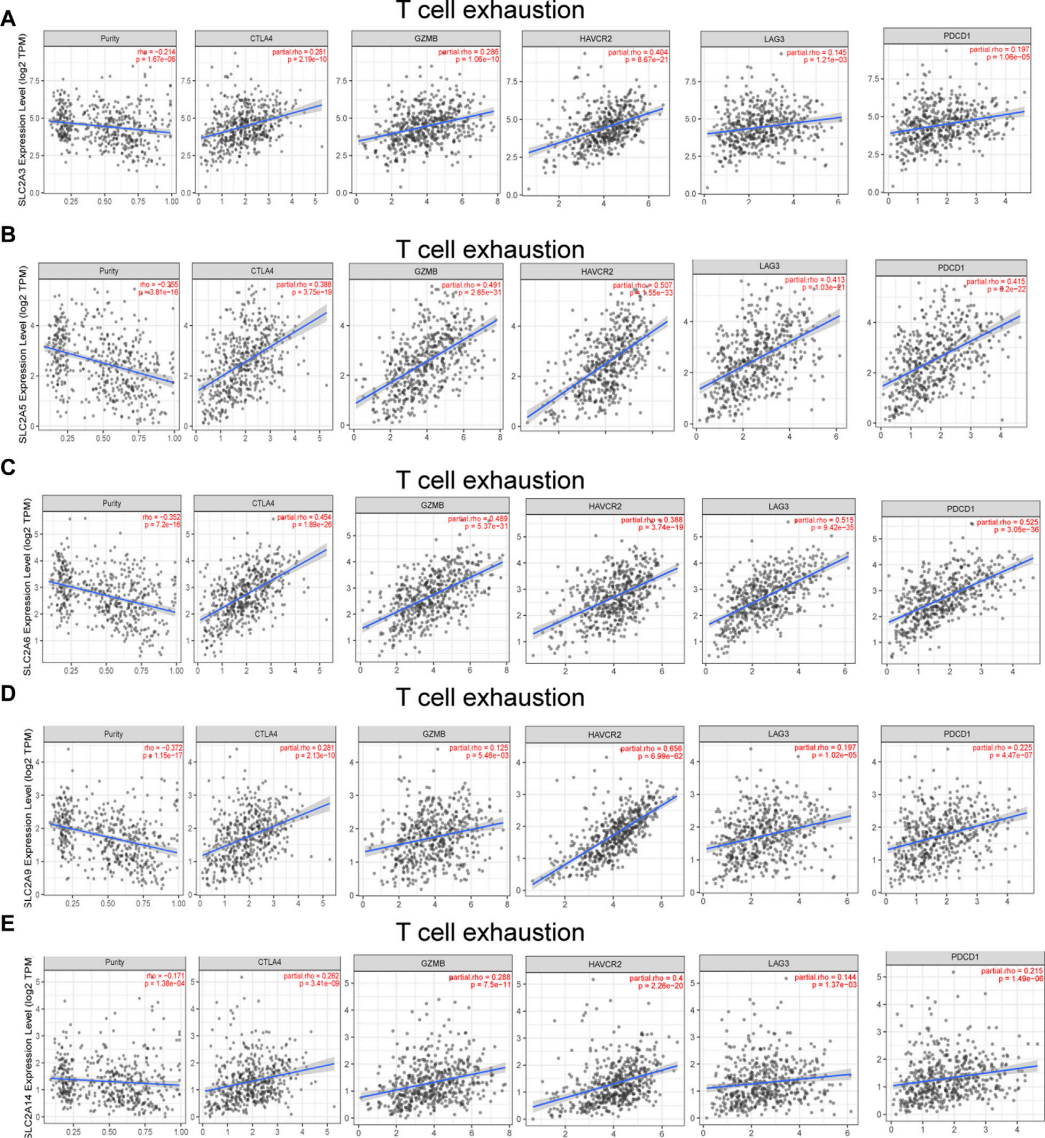
*SLC2As* expression has significant relations with infiltration of T cell exhaustion in LUAD. **(A)** *SLC2A3*. **(B)** *SLC2A5*. **(C)** *SLC2A6*. **(D)** *SLC2A9*. **(E)** *SLC2A14*. TCE marker genes include PDCD1, CTLA4, LAG3, HAVCR2, and GZMB.

Different illness states can stimulate immune cells (Fridlender et al., 2009; Murray, 2017). In LUAD patients, we attempted to determine whether *SLC2As* are correlated with the marker genes of different immune cell types, such as CD8^+^ T cells, monocytes, T cells (general), M1 and M2 macrophages, B cells, neutrophils, tumor-associated macrophages (TAMs), natural killer cells, and DCs. We also examined T cells with various subtypes, including fatigued T cells and T-helper type 1 (Th1), Th17, Th2, and Treg (Supplementary Material S4). *SLC2As* substantially linked with the majority of marker genes of various types of immune cells and diverse functional T cells in LUAD after tumor purity-correlated modifications. *SLC2A3*, *SLC2A5*, and *SLC2A14* expressions were all shown to be substantially linked with M2 macrophages and monocytes. Additionally, *SLC2A6* expression was highly connected with M2 macrophages, M-DC, monocytes, and Treg cells, while *SLC2A9* expression was significantly correlated with T cells, Th1 cells, and monocyte infiltration (Supplementary Material S2; Figure 4). Expressions of *SLC2A6* and *SLC2A9* were positively correlated with TAMs (Supplementary Material S4). These findings show how these molecules are involved in enlisting and activating various immune cell subtypes to take part in the pathogenesis of LUAD. Additionally, there was a positive correlation between the levels of *SLC2A3*, *SLC2A5*, *SLC2A6*, *SLC2A9*, and *SLC2A14* and markers of T cell exhaustion (TCE) (PDCD1, CTLA-4, LAG3, HAVCR2, GZMB), identifying that the elevated level of these molecules implied the presence of TCE in LUAD (Figure 8).

### The mRNA expression in clinical samples of LUAD

**FIGURE 9:**
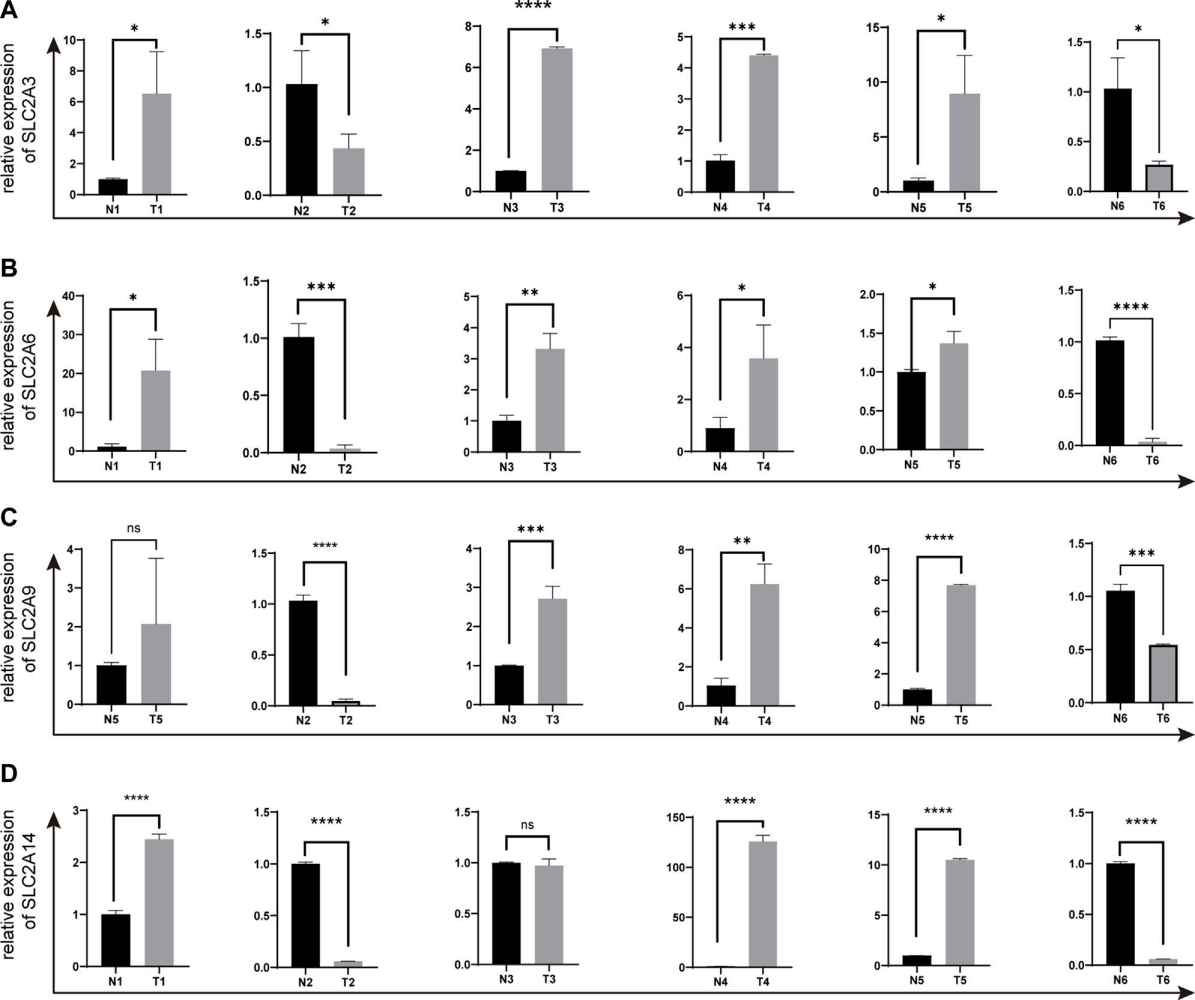
The expressions of *SLC2A3*, *SLC2A6*, *SLC2A9* and *SLC2A14* in clinical LUAD samples were validated by q-PCR **(A)** *SLC2A3*. **(B)** *SLC2A6*. **(C)** *SLC2A9*. **(D)** *SLC2A14*. T, Tumor. N, Normal adjacent tissues.

We selected *SLC2A3*, *SLC2A6*, *SLC2A9*, and *SLC2A14* as verified targets in our clinical samples since prior research has demonstrated higher expression of *SLC2A1* and *SLC2A5* in LUAD patients. Patients numbers 2 and 6 had lower levels of *SLC2A3*, *SLC2A6*, *SLC2A9*, and *SLC2A14* than the surrounding normal tissue. However, patient number 1 had elevated levels of *SLC2A3*, *SLC2A6*, and *SLC2A14*, whereas patient number 3 had elevated levels of *SLC2A3*, *SLC2A6*, and *SLC2A9*. In patient number 4 and 5, elevated levels of *SLC2A3*, *SLC2A6*, *SLC2A9*, and *SLC2A14* were discovered (As shown in Figure 9; Supplementary Material S5).

## Discussion

Three classifications have been established for GLUTs (Hardie and Ashford, 2014). Class 1, which includes GLUTs 1-4 and GLUT-14 and contains glucose as its substrate, has a substantially lower affinity for fructose (Mueckler and Thorens, 2013). GLUT-5, GLUT-7, GLUT-9, and GLUT-11 are all members of class 2. Fructose serves as GLUT-5’s primary substrate, and it has a higher affinity for it than for glucose (Tatibouët et al., 2000). While GLUT- 7 and GLUT-11 have the same affinity for glucose as fructose (Manolescu et al., 2007; Cheeseman, 2008) GLUT-9 transports urate along with fructose in the physiological range (Cheeseman, 2008; So and Thorens, 2010). Class 3, which consists of GLUT-6, GLUT-8, GLUT-10, GLUT-12, and GLUT-13, is challenging to characterize. The existing evidence indicated that GLUT-12 transports glucose (Macheda et al., 2005) and GLUT-13 transports myo-inositol (Holman, 2020). However, it is still unknown what the other members substrates are (Holman, 2020). *SLC2As* have reportedly been implicated in multiple diseases, including cancer. This study is the first comprehensive biological analysis of the 14 *SLC2A* genes in LUAD patients.

The results of the researchers’ numerous studies on *SLC2As* expressions in LUAD at the mRNA and protein levels are inconsistent or even conflicting. One study identifies *SLC2A1* may be a crucial glucose transporter in LUAD patients as *SLC2A1* mRNA and protein levels are elevated in LUAD patients, and its overexpression is a potential indicator for lower differentiated tumor grade and poor prognosis (Younes et al., 1997a; Minami et al., 2002; Maki et al., 2013; Chai et al., 2017; Weng et al., 2018a; Guo et al., 2020). However, another study demonstrates that GLUT-1 protein expression is absent in healthy lung tissues but positive in 14 of 24 adenocarcinomas and that the levels of expression are correlated with greater tumor sizes, less differentiation of the tumor, and positive lymph node metastases (Ito et al., 1998). LUAD patients did not exhibit altered *SLC2A3* and *SLC2A5* expressions at the mRNA level when compared to healthy donors. However, compared to the primary tumor, lung cancer metastases have higher levels of *SLC2A3* and *SLC2A5*, highlighting their significance in tumor metastasis (Kurata et al., 1999). Another study uses immunohistochemical analysis to show that GLUT-3 and GLUT-5 are expressed at the same levels as those of healthy lung tissues (Holman, 2020), while GLUT-4 expression is positive and GLUT-2, GLUT-3, and GLUT-5 are negative in LUAD patients (Brown et al., 1999). Regarding GLUT-3 protein levels, numerous investigations show that they are absent in healthy lung tissues (Younes et al., 1997b) and increased in non-small cell lung cancer (de Geus-Oei et al., 2007; Suzawa et al., 2011). Compared to healthy tissues, the level of *SLC2A5* mRNA is higher in LUAD tissues. Additionally, a worse prognosis is connected to *SLC2A5* overexpression (Weng et al., 2018a; Weng et al., 2018b). GLUT-3, GLUT-4, and GLUT-5 have been discovered to be overexpressed in LUAD patients at the protein level (Younes et al., 1997b; Flavahan et al., 2013; Chai et al., 2017). In a different study, GLUT-2, GLUT-3, GLUT-4, GLUT-5, GLUT-6, GLUT-9 expression levels were tested in lung adenocarcinoma and only GLUT-3 is positive in 1 of 8 lung adenocarcinoma patients (Godoy et al., 2006). Neither GLUT-3 nor GLUT-4 is positively stained in normal lung epithelia while lung cancers have elevated levels of *SLC2A3* and *SLC2A4* (Ito et al., 1998). H1299 (NSCLC cell line) has high *SLC2A12* mRNA expression (Zawacka-Pankau et al., 2011), while lung cancer cell line A549 has high levels of GLUT-12 expression (Pujol-Gimenez et al., 2015). The predictive value of the *SLC2As* in LUAD patients has also been examined. Increased GLUT-3 levels are a potential indicator of a poor prognosis and a biomarker for lower tumor differentiation in Stage I non-small cell lung cancer patients, highlighting the significance of GLUT-3 for early diagnosis and prognostic accuracy (Younes et al., 1997b; Maki et al., 2013). According to a different study, patients with NSCLC who have high levels of the mRNAs *SLC2A1*, *SLC2A2*, *SLC2A3*, *SLC2A4*, *SLC2A5*, *SLC2A6*, *SLC2A7*, *SLC2A9*, *SLC2A11*, and *SLC2A14* have a considerably worse overall survival (Du et al., 2020). Downregulated levels of *SLC2A10* in lung cancer indicate a poor prognosis and increased aggressiveness of tumors, suggesting that *SLC2A10* may play a crucial role in the progression of cancer (Jian et al., 2021).

In our study, we show that transcriptional levels of *SLC2A1*, *SLC2A5* are upregulated and those of *SLC2A3*, *SLC2A6*, *SLC2A9*, *SLC2A14* are downregulated in LUAD patients, supporting the role of *SLC2A1*, *SLC2A5* as oncogenes and *SLC2A3*, *SLC2A6*, *SLC2A9*, *SLC2A14* as tumor suppressor genes. Inhibiting GLUT-1, GLUT-5, GLUT-6, and GLUT-9 may be used as a therapy for LUAD patients as a result of the increased protein levels of these enzymes that were shown in our study. *SLC2As*’ mismatched mRNA and protein levels in LUAD patients could be explained by changes in turnover (transcription and degradation), as well as post-transcriptional and post-translational modifications like phosphorylation, acetylation, ubiquitination, and methylation, as well as sumo of the *SLC2As* or GLUTs. Furthermore, the upregulation of GLUT-1, GLUT-5, GLUT-6, and GLUT-9 suggests that fructose and glucose may both play important roles in LUAD metabolism, supporting the development of blockades for these molecules as potential new medications or biomarkers for the treatment or detection of LUAD. Re-examining the mRNA expression of *SLC2A3*, *SLC2A6*, *SLC2A9*, and *SLC2A14* revealed that 2 of 6 patients had lower levels of these genes than those predicted by RNA-seq methods in LUAD samples. The results from the other four LUAD patients were different, showing higher levels of *SLC2A3*, *SLC2A6*, *SLC2A9*, and *SLC2A14* in LUAD patients. The differing experimental platforms could be the cause of the varied expression levels between RNA-seq and q-PCR. Nearly the whole exon region of a gene is covered by RNA-seq, which also considers the expression levels of each exon region. However, q-PCR does not account for the entire length of the gene when designing primers or amplifying.

Because patients in the same phase might have quite different clinical outcomes, even at an early stage, it is important to identify novel tumor biomarkers in order to accurately estimate patient prognosis and predict responsiveness to a specific therapy. The next step is to assess if tumor stages can be identified based on *SLC2As* expression levels. A lower differentiation grade tumor is implied by overexpression of *SLC2A1*, *SLC2A5*, *SLC2A10*, *SLC2A11*and lower levels of *SLC2A3*, *SLC2A6*, *SLC2A9*, *SLC2A12*, and *SLC2A14*, emphasizing the possibility of these molecular roles for predicting the course of the tumor. Interestingly, the expression levels of *SLC2A1*, *SLC2A3*, *SLC2A5*, *SLC2A6*, *SLC2A7*, *SLC2A9*, *SLC2A10*, *SLC2A11*, *SLC2A12*, and *SLC2A14* all differed in stage 1 compared to normal tissues, strongly proposing these molecules as possible markers for the early diagnosis of LUAD. Secondly, we establish the importance of low levels of *SLC2A1*, *SLC2A2*, *SLC2A3*, *SLC2A4*, *SLC2A5*, *SLC2A9*, *SLC2A14* and high levels of *SLC2A10*, *SLC2A12*, and *SLC2A13* as biomarkers for favourable prognosis. Results for *SLC2A1*, *SLC2A3*, *SLC2A5*, and *SLC2A10* are in line with earlier investigations. It is intriguing that individuals with LUAD who had high levels of *SLC2A9* and *SLC2A14*, which were previously identified as suppressor genes in our investigations, have lower survival rates. We think that these molecules may be the source of the resistance to chemotherapy and radiation. Analyses using the LASSO technique are used to investigate the most important factors. *SLC2A1*, *SLC2A7*, and *SLC2A11* are three *SLC2As* that have been discovered as possible predictive biomarkers. *SLC2A11* is an indicator of a favorable outcome in LUAD, but high expression of *SLC2A1* and *SLC2A7* is a characteristic of an adverse prognosis. The combination of *SLC2A1*, *SLC2A7*, and *SLC211* is also supported by the ROC curve as a possible biomarker for 1-year, 3- year, and 5-year survival. In conclusion, *SLC2As* may someday serve as critical clinical markers for LUAD patients, allowing for earlier identification, more precise risk classification, and individualized survival prediction.

The role of epigenetic regulators, such as DNA methylation, on *SLC2As* deregulation, was identified using the UALCAN database to investigate the upstream mechanisms causing the abnormal *SLC2As* expression. Focusing on how epigenetic alteration affects cancer etiology, both suppressor gene suppression caused by excessive methylation in gene promoters and oncogene activation or chromosomal instability caused by widespread hypomethylation can occur in malignancies (Merlo et al., 1995; Rosty et al., 2002; Gaudet et al., 2003). Particularly, the development of DNA methylation liquid biopsy has made early cancer diagnosis and treatment recommendations possible (Brikun et al., 2018; Shen et al., 2018; Zhao et al., 2018). In our research, hypomethylation occurs on oncogenes including *SLC2A1*, *SLC2A5*, *SLC2A7*, and *SLC2A11*. Additionally, hypermethylation of tumor suppressor genes, such as *SLC2A3* and *SLC2A14*, is observed in LUAD patients, suggesting that DNA methylation may be the cause of the aberrant expression of *SLC2As* in LUAD. There is a lot of interest in treatments that target epigenome alterations, such as DNA methyltransferases (DNMTs). DNMTs have been used as anti-cancer drugs, specifically as potential chemotherapy or radiotherapy sensitizers, to increase treatment efficacy (Gravina et al., 2010). Given the significance of DNA methylation affects *SLC2As*, DNMTs have a potential for correcting these aberrant *SLC2As* expressions in LUAD. To determine the level of DNA methylation in LUAD, future studies will need to carefully implement animal and cell line research. These results will lay the groundwork for DNA methylation as sensitive prognostic, predictive biomarkers, and therapeutic targets in LUAD.

What role did the aberrant *SLC2As* play in LUAD pathogenesis? Due to the co-expressed genes’ enrichment in GO and KEGG pathways associated with immune cells, we concentrate on the relationship between *SLC2As* and immunological signatures. Recently, numerous tumors have been successfully treated using a variety of immunotherapy techniques, including immune checkpoint blockades (PD-1, CTLA4), cellular therapy, and therapeutic vaccinations, including LUAD (Del Paggio, 2018; Christofi et al., 2019). However, not every immunotherapy is successful in treating patients due to patients’ limited tumor immunity and the heterogeneity of checkpoint inhibitors (Sasidharan Nair and Elkord, 2018; Liu et al., 2020). In addition, tumor cells are prone to switch from oxidative metabolism to glycolysis and lactic acid fermentation even in normal conditions. The metabolic alteration leads to hypoxia, production of acid, poor in nutrients and abundance of immune-modulatory metabolites, consequently contributing to the resistance to immunotherapy (Ramapriyan et al., 2019). Therefore, we aim to better understand the connection between *SLC2As* and immune cell infiltration in LUAD, which will aid in identifying patients who may benefit from immune therapy. The alteration of the tumor microenvironment caused by circulating monocytes and macrophages, which are drawn to the tumor, promotes the growth of malignancies (Chanmee et al., 2014). Macrophages are generally classified into two subpopulations, classically activated pro-inflammatory macrophages (M1) and alternatively activated macrophages (M2). M2 macrophages are involved in promoting the growth and invasion of lung cancer cell, while M1 mac rophages inhibit lung cancer cell proliferation and activity *via* increasing cancer cell chemical sensitivity and decreasing angiogenesis (Yuan et al., 2015). It is interesting to note that in LUAD patients, M2 and monocyte expression levels are favorably linked with *SLC2A3* and *SLC2A5* expression levels. Th1, monocytes, and T cells had a positive correlation with *SLC2A6* levels. M2, TAM, M-DC, monocytes, and Treg cells all exhibit favorable correlations with *SLC2A9* expression levels. In LUAD patients, *SLC2A14* expression is favorably linked with M2, monocytes, and TAM. These findings reveal that members of the *SLC2A* family may contribute to carcinogenesis by controlling M2 polarization and drawing monocytes and Tregs to tumor locations. However, more research into the underlying mechanism is still required. Additionally, it has been suggested that TCE plays a role in various malignancies and chronic infections, a condition marked by a decline in T cell activity, persistent expression of inhibitory receptors, and a transcriptional state distinct from that of functioning effector or memory T cells and inhibiting pathways elevated in exhaustion can reverse the dysregulated state and reinvigorate immune responses (Wherry and Kurachi, 2015). According to our findings, expression of *SLC2A3*, *SLC2A5*, *SLC2A6*, *SLC2A9*, and *SLC2A14* is positively correlated with the TCE marker gene, suggesting that these molecules may reflect the presence of TCE in LUAD. All these findings supported the use of *SLC2A3*, *SLC2A5*, *SLC2A6*, *SLC2A9*, and *SLC2A14* as therapeutic targets and biomarkers for evaluating the effectiveness of immunotherapy.

Finally, we suggest multiple pathways in which *SLC2As* were involved in LUAD, including *SLC2A1*/BUB1B/mitotic cell cycle, *SLC2A5*/CD86/negative immune system process regulation, *SLC2A6*/PLEK/lymphocyte activation, and *SLC2A9*/CD4/cytokine production regulation.

The results that an increased level of GLUT-1 is positively correlated with F-18 fluorodeoxyglucose (FDG) uptake indicate the involvement of GLUT-1 in the accumulation of FDG in LUAD patients (Brown et al., 1999; Gu et al., 2006; Wink et al., 2017; Vanhove et al., 2019). However, as confounders in PET positron emission tomography-computer tomography (PET-CT) analysis, inflammatory pseudotumor, tuberculoma, and organizing pneumonia may present a similar picture with lung malignancies. We hypothesize that the combination of GLUT-1, GLUT-5, GLUT-6, and GLUT-9 may distinguish the inflammation (necrosis) lesions from malignancies in light of the increased protein expression of GLUT-1, GLUT-5, GLUT-6, and GLUT-9 in LUAD patients in our study. However, the hypothesis needs further investigation.

GLUT inhibitors have been used in numerous studies to treat NSCLC. A GLUT-1 inhibitor called WZB117 prevents the proliferation of the NSCLC cancer cells (H1299 and A549) (Liu et al., 2012). Oxime-based GLUT-1 inhibitors reduce the proliferation of the cancerous H1299 cell line (Tuccinardi et al., 2013). The GLUT-2 inhibitor quercetin lowers the risk of developing lung cancer (Rastogi et al., 2007). Small interfering RNA (siRNA) for GLUT-1 increases NSCLC cell susceptibility to gefitinib treatment (Suzuki et al., 2018). Anti-GLUT-1 antibodies reduce non-small cell lung cancer cell growth by 50% (Rastogi et al., 2007). However, the findings that both glucose and fructose are energy sources in LUAD remind us that cancer cells can switch to a different option for their energy requirements, so we recommend that the best course of action may be to completely inhibit GLUT-transporters. Blocking GLUT-1, GLUT-5, GLUT-6, and GLUT-9 may display beneficial efficiency in LUAD therapy. A better understanding of the universal expression profiles of GLUTs and the tissue distribution of GLUTs will pave the way for transforming the GLUT inhibitors into clinical application in LUAD.

## Conclusion and outlooks

The prognosis of patients with LUAD can be correctly stratified with the use of a thorough examination of their metabolic profiles. Our findings might suggest *SLC2As* as biomarkers that could help distinguish between low-risk LUAD patients and advanced LUAD patients. Moreover, *SLC2As* could serve as prognostic signatures to predict 1, 3, and 5 year survival in LUAD patients. *SLC2As* are abnormally expressed in LUAD due to DNA methylation, and *SLC2As* that are dysregulated promote LUAD by changing the immunological microenvironment. We also suggest several pathways that could be involved in LUAD, including *SLC2A1*/BUB1B/mitotic cell cycle, *SLC2A5*/CD86/negative immune system process regulation, *SLC2A6*/PLEK/lymphocyte activation, and *SLC2A9*/CD4/cytokine production regulation. These findings open the door to the use of these SLC2A molecules as LUAD biomarkers and treatment targets.

Due to their propensity to mimic other inflammatory diseases, cancers diagnosed by PET-CT were likewise less sensitive and specific. GLUTs should work together as radiotracers to efficiently image LUAD tumors and provide early identification, accurate tumor grading, and prognosis prediction.

The future trend will be to completely inhibit GLUT-1, GLUT-5, GLUT-6, and GLUT-9 as therapeutic targets. Our findings revealed that LUAD uses fructose (a GLUT-5 and GLUT-9 substrate) as a different type of energy source. Since GLUT-5 and GLUT-9 are not universally expressed as GLUT-1, blocking the transport of fructose may be an appealing method to inhibit the metabolism of glucose. In other words, it is important to consider the high specificity. We advise exosomes or nanoparticles loaded with GLUT inhibitors as a viable option that might be safely and conveniently delivered to combat LUAD.

The GLUT function has previously been studied using cell lines, but this method has the disadvantage of not compressing other tissue-matrix components. To shed light on the significance of glucose carriers in LUAD patients, it is necessary to conduct these experimental and clinical trial tests in *SLC2A* conditional knockout mice, which enable GLUT knocking out in specific cells. Another related problem is that we still do not fully comprehend how *SLC2As* regulate various immune cell subtypes, such as TCE, M2, T cells, and monocyte in LUAD.

## Data Availability

The original contributions presented in the study are included in the article/Supplementary Material, further inquiries can be directed to the corresponding author.
