# Supplementary material for: SLC2As as diagnostic markers and therapeutic targets in LUAD patients through bioinformatic analysis
Source: Front Pharmacol. 2022 Nov 28;13:1045179. doi: 10.3389/fphar.2022.1045179 (PMC9742449; doi:10.3389/fphar.2022.1045179)
Supplement: Supplementary file 4 [file DataSheet1.docx]

**Supplementary material S2**

**
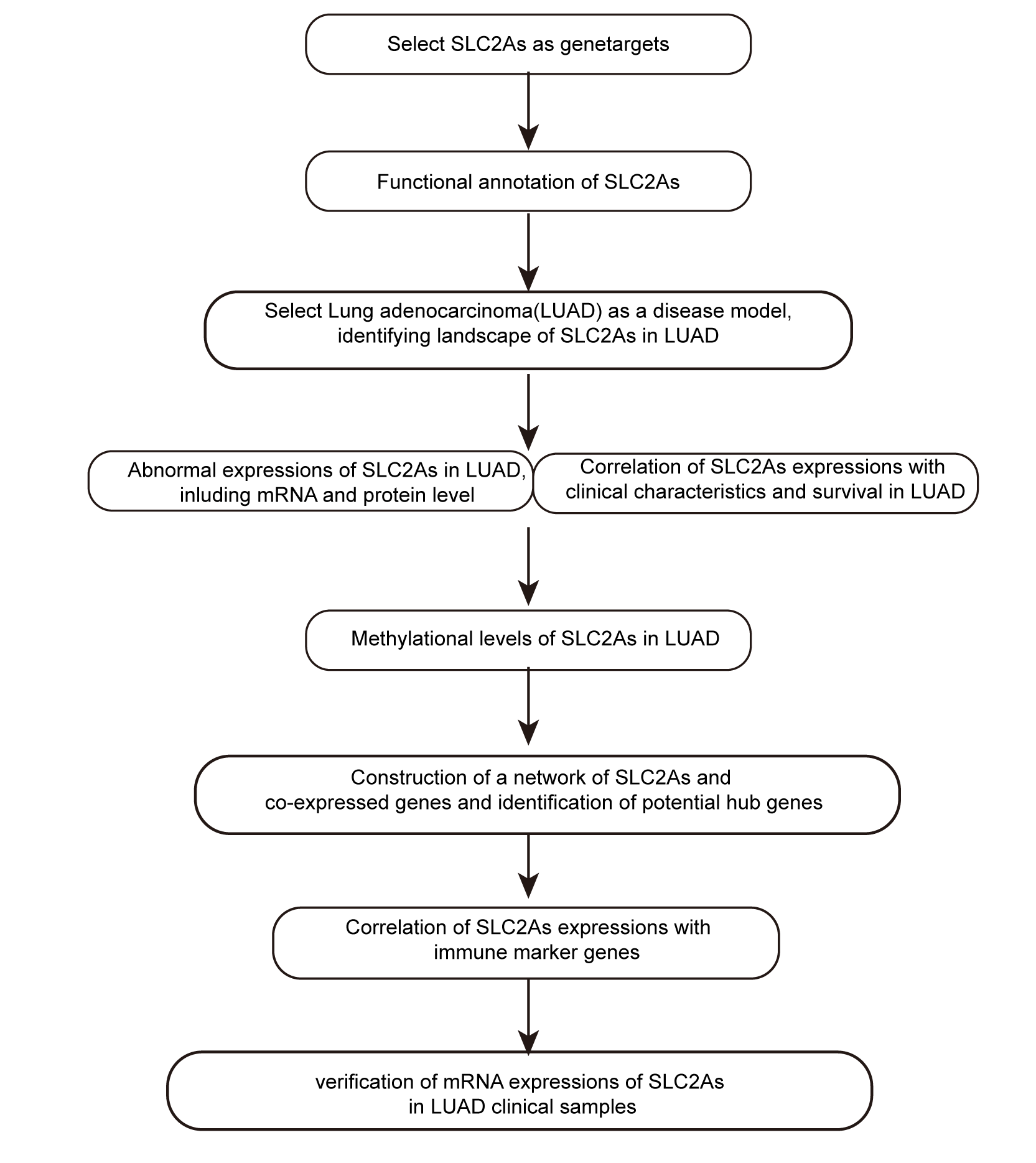
**

**Figure 1** Flowchart for profiling *SLC2As* of LUAD

**
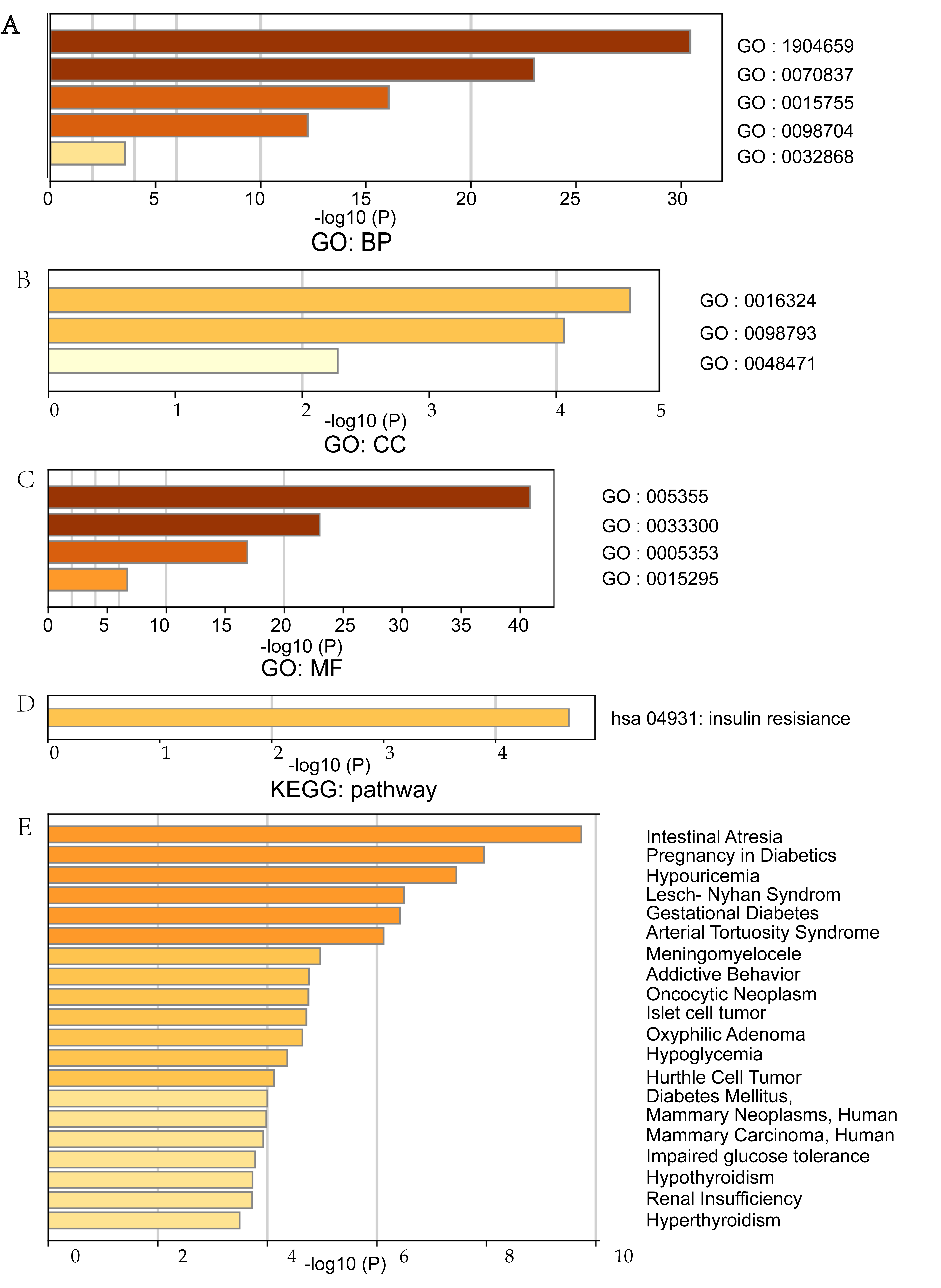
**

**Figure 2** GO annotations and Kyoto Encyclopedia of Genes and Genomes (KEGG) pathway analyses of *SLC2As*. (A) biological process. (B)Cellular components. (C) Molecular functions. (D) Pathway analyses using the KEGG database. (E) diseases associated with *SLC2As*.


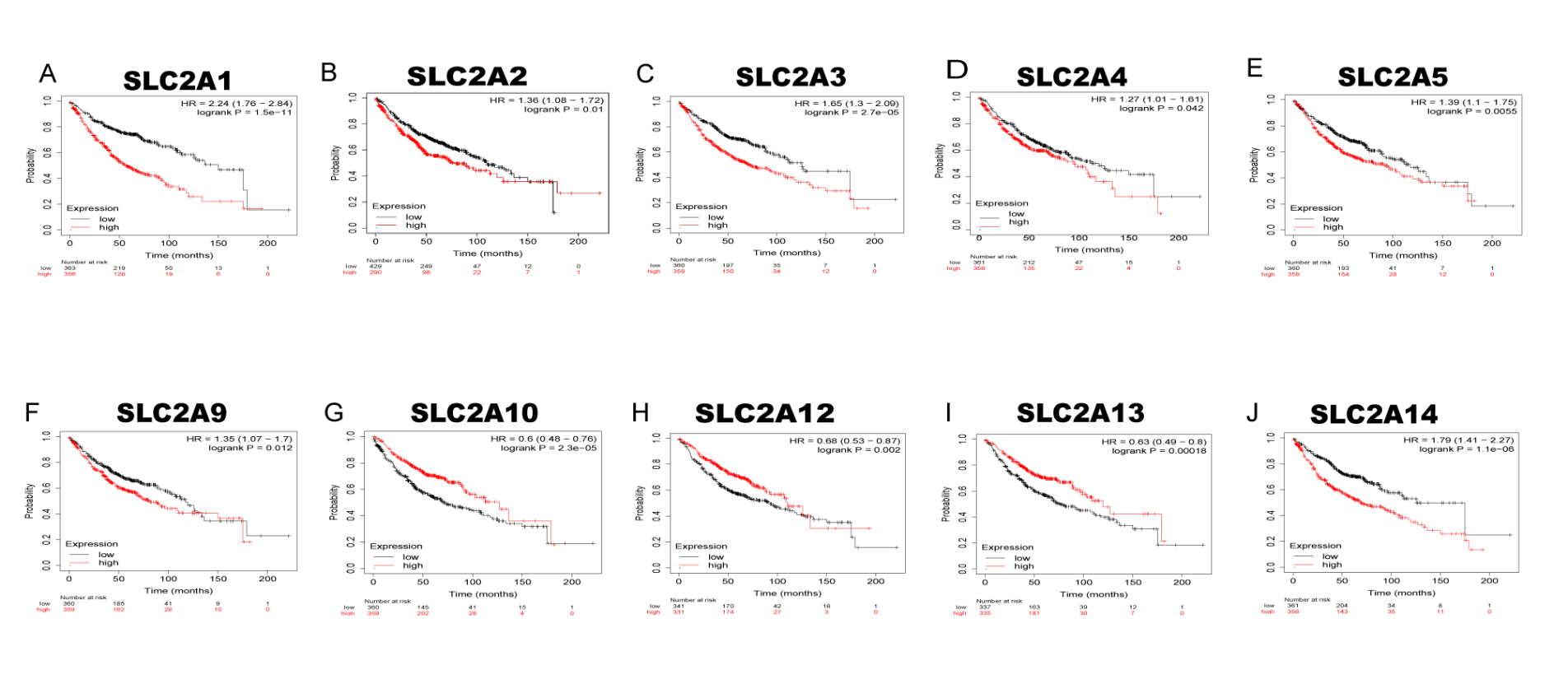


**Figure 3** Prognostic value of mRNA expression of distinct *SLC2As* in LUAD (Kaplan-Meier Plotter). Lower mRNA expressions of *SLC2A1, SLC2A2,*

*S LC2A3, SLC2A4, SLC2A5, SLC2A9, SLC2A14* and higher mRNA level of *SLC2A10, SLC2A12, SLC2A13* were significantly associated with favorable Overall Survial (OS) of LUAD (A-J).


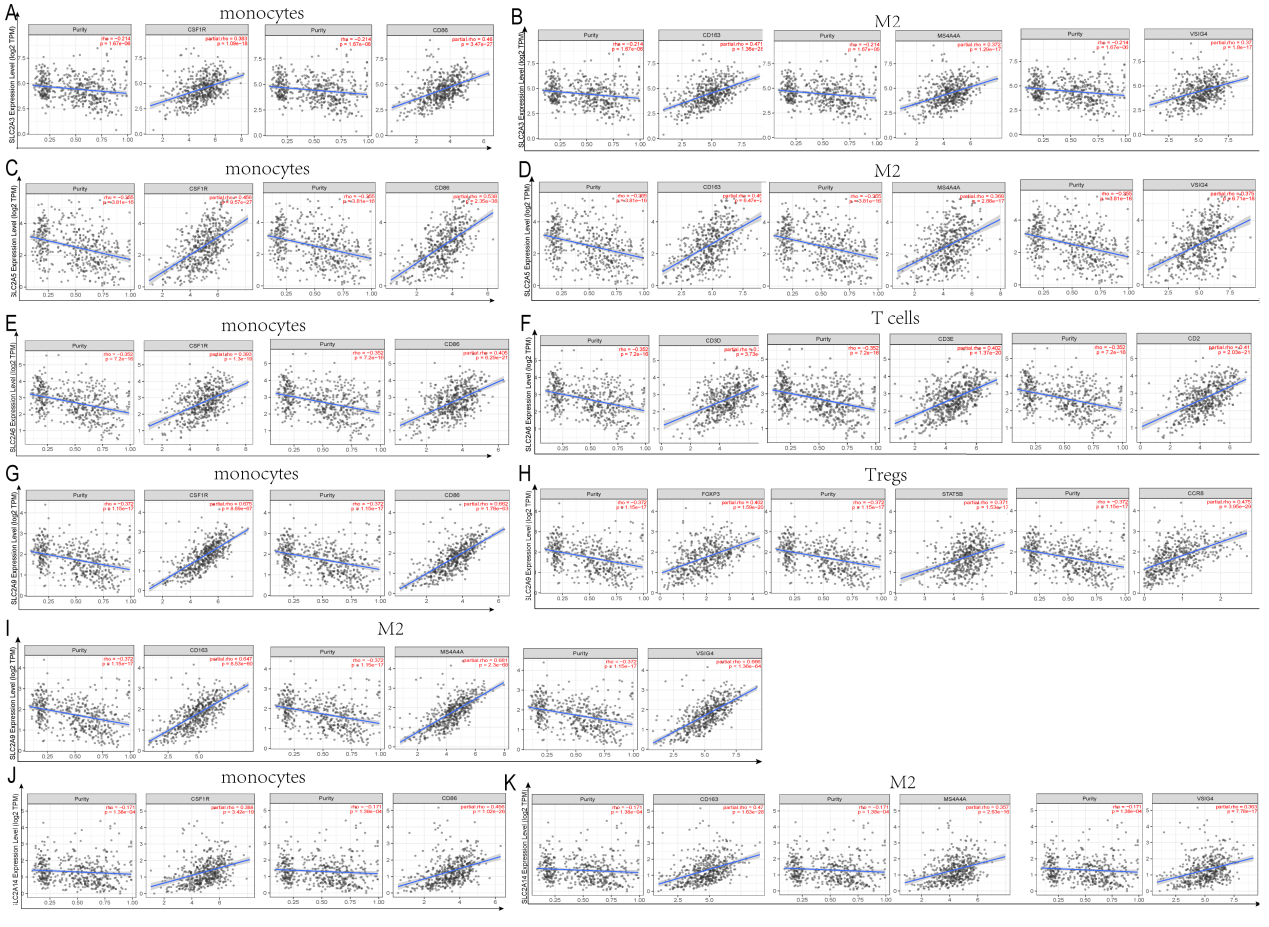
**Figure 4** *SLC2As* expression has significant correlations with infiltration of immune cells and macrophage polarization in LUAD. (A-D, J-K) *SLC2A3, SLC2A5, SLC2A14* expressions were all in significantly positive correlation with infiltration of monocytes and M2 macrophages in LUAD. (E-F) *SLC2A6* expression was in significantly positive correlation with infiltration of monocytes and T cells in LUAD. (G-I) *SLC2A9* expression was in significantly positive correlation with infiltration of monocytes, Tregs and M2 macrophages in LUAD.

**
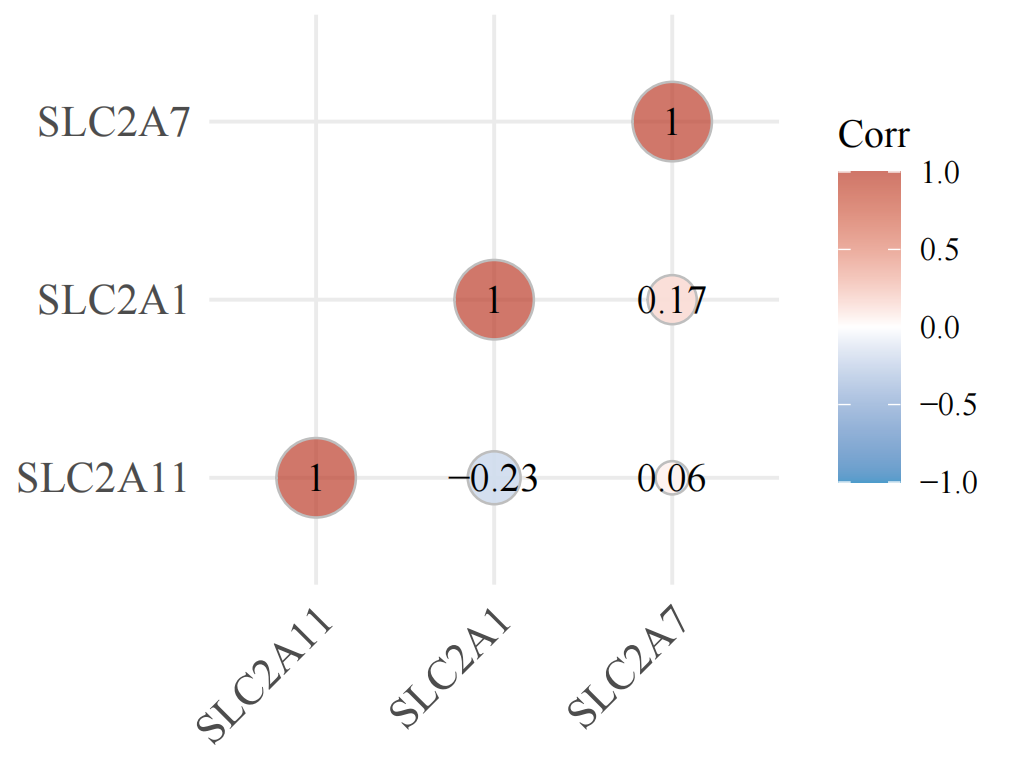
Figure 5** A heatmap of the correlation among *SLC2A1*, *SLC2A7*, and *SLC2A11*.The abscissa and ordinate represent genes, different colors represent different correlation coefficients (blue represents positive correlation whereas red represents negative correlation), the darker the color, the stronger the relation. Asterisks (*) stand for significance levels, ** p < 0.01, * p < 0.05.
